# Genetic variation in South Indian castes: evidence from Y-chromosome, mitochondrial, and autosomal polymorphisms

**DOI:** 10.1186/1471-2156-9-86

**Published:** 2008-12-12

**Authors:** WS Watkins, R Thara, BJ Mowry, Y Zhang, DJ Witherspoon, W Tolpinrud, MJ Bamshad, S Tirupati, R Padmavati, H Smith, D Nancarrow, C Filippich, LB Jorde

**Affiliations:** 1 Department of Human Genetics, University of Utah, Salt Lake City, UT 84112, USA; 2 Schizophrenia Research Foundation, Chennai, 600101, India; 3 Queensland Centre for Mental Health Research, Wacol, Brisbane, 4076, Australia; 4 Queensland Institute of Medical Research, Herston, Brisbane, 4006, Australia; 5 Department of Pediatrics, University of Washington School of Medicine, Seattle, WA 98195, USA

## Abstract

**Background:**

Major population movements, social structure, and caste endogamy have influenced the genetic structure of Indian populations. An understanding of these influences is increasingly important as gene mapping and case-control studies are initiated in South Indian populations.

**Results:**

We report new data on 155 individuals from four Tamil caste populations of South India and perform comparative analyses with caste populations from the neighboring state of Andhra Pradesh. Genetic differentiation among Tamil castes is low (R_ST _= 0.96% for 45 autosomal short tandem repeat (STR) markers), reflecting a largely common origin. Nonetheless, caste- and continent-specific patterns are evident. For 32 lineage-defining Y-chromosome SNPs, Tamil castes show higher affinity to Europeans than to eastern Asians, and genetic distance estimates to the Europeans are ordered by caste rank. For 32 lineage-defining mitochondrial SNPs and hypervariable sequence (HVS) 1, Tamil castes have higher affinity to eastern Asians than to Europeans. For 45 autosomal STRs, upper and middle rank castes show higher affinity to Europeans than do lower rank castes from either Tamil Nadu or Andhra Pradesh. Local between-caste variation (Tamil Nadu R_ST _= 0.96%, Andhra Pradesh R_ST _= 0.77%) exceeds the estimate of variation between these geographically separated groups (R_ST _= 0.12%). Low, but statistically significant, correlations between caste rank distance and genetic distance are demonstrated for Tamil castes using Y-chromosome, mtDNA, and autosomal data.

**Conclusion:**

Genetic data from Y-chromosome, mtDNA, and autosomal STRs are in accord with historical accounts of northwest to southeast population movements in India. The influence of ancient and historical population movements and caste social structure can be detected and replicated in South Indian caste populations from two different geographic regions.

## Background

The origins and genetic affinities of India's populations have been debated extensively [1-6]. Archaeological studies document human occupation of the subcontinent from the lower Paleolithic through the Neolithic, including a flourishing ancient civilization in the Indus Valley [7]. The historical record documents an influx of Vedic Indo-European-speaking immigrants into northwest India starting at least 3500 years ago [8-11]. These immigrants spread southward and eastward into an existing agrarian society dominated by Dravidian speakers [12]. With time, a more highly-structured patriarchal caste system developed [7,9,10]. India is now broadly characterized by Indo-European (e.g. Hindi, Urdu, and Punjabi) speaking populations found in the central and northern regions and by Dravidian (e.g. Tamil, Telugu, and Kannada) speaking populations in the southern and southeastern regions. The extent to which ancient and contemporary migrations, and the more recent inception of a hierarchical caste system, have influenced the genetic composition of modern Indian populations remains controversial.

A number of studies have addressed the genetic contribution of other Eurasian populations to Indian caste and tribal populations [1-3,6,13-18]. They have arrived at somewhat different conclusions regarding the origins of castes, their relationships to each other, and their relationship to populations outside India. These discordances can be attributed, in part, to differences in sampling strategies and the varied effects of gene flow between the typically endogamous castes and tribes [14,19-21].

Several trends regarding the origin and affinities of Indian populations have emerged. The predominantly south and east Asian mtDNA haplogroup M is found in more than half of individuals from a wide sampling of castes [5,6,13,22] and is nearly fixed in some Austro-Asiatic tribal populations [6]. This haplogroup is uncommon in western European populations [23,24]. In contrast, some paternally-inherited Y-chromosome lineages are more closely related to lineages originating in central Asians and Europeans [1,13,25,26]. Genetic distances estimated from autosomal polymorphisms have typically demonstrated that caste populations tend to occupy a position intermediate between European and East Asian populations [8,27-29].

The genetic affinities among the more than 2000 extant caste populations of India, however, are complex. Genetic distances between caste populations from the state of Andhra Pradesh, India, are correlated with differences in caste rank, suggesting that endogamy and differential inter-caste gene flow influences genetic structure [30]. Several studies have found a similar pattern, [31-33] but others have not [6,34]. Higher rank castes may show closer affinity to European populations than do other caste populations [13]. Recent Y-chromosome data suggest a higher affinity between tribal populations and castes of lower rank [35].

These results support historical accounts of nomadic pastoralists from central and northwestern Eurasia integrating with existing local populations, and either introducing a system of social stratification or becoming members of the existing upper castes [8,9,35]. Yet, the occurrence of Y-chromosome haplogroups L, H, R2, and R1a in both caste and isolated tribal populations suggests much of the existing Indian population structure is very old [5]. Additionally, the high diversity of Y haplogroups R1a1 and R2 in both South Indian and Indus valley populations has led to the suggestion that there is little, if any, genetic influence from other Eurasians on the castes of South India [3].

A broad study of 24 castes from various locations throughout India concluded that genetic data were not congruent with "sociocultural" affinities due to high rates of gene flow [6]. Yet, this study and others [1,36] have suggested a clinal (north to south) contribution of central Asian Y-chromosomal lineages to caste populations. Due to well-established clines in gene frequencies across India, especially in the north-south direction, [2,34,36] comparisons of castes from different geographic locations can conflate clinal variation with variation that may exist between local caste groups. Therefore, it is important to obtain large, carefully chosen samples from the same geographic locale to determine whether previous results [13] indicating caste-related genetic structure can be replicated in other regions of India [37]. Additionally, because single linkage groups such as the non-recombining region of the Y-chromosome or the mtDNA genome may be strongly influenced by genetic drift or selection, the use of a large number of independent autosomal polymorphisms can greatly improve the reliability of estimates of population relationships.

In this study we analyze four castes of different rank sampled from Tamil Nadu, the southern-most state of the Indian subcontinent. The genetic relationship among the Tamil castes, their relationship to castes from the neighboring state of Andhra Pradesh, and their affinity to other Eurasian populations are examined using Y-chromosome, mtDNA, and autosomal polymorphisms. We show that the genetic affinities between Indian castes from Tamil Nadu and other Eurasians are broadly congruent with patterns observed previously for castes from Andhra Pradesh. These results strengthen the conclusions drawn from our previous analyses regarding caste relationships in South India and suggest reproducible patterns regarding the genetic influence of ancient and historical events on the Indian caste system.

## Results

### Y-chromosome haplogroups

**Figure 1:**
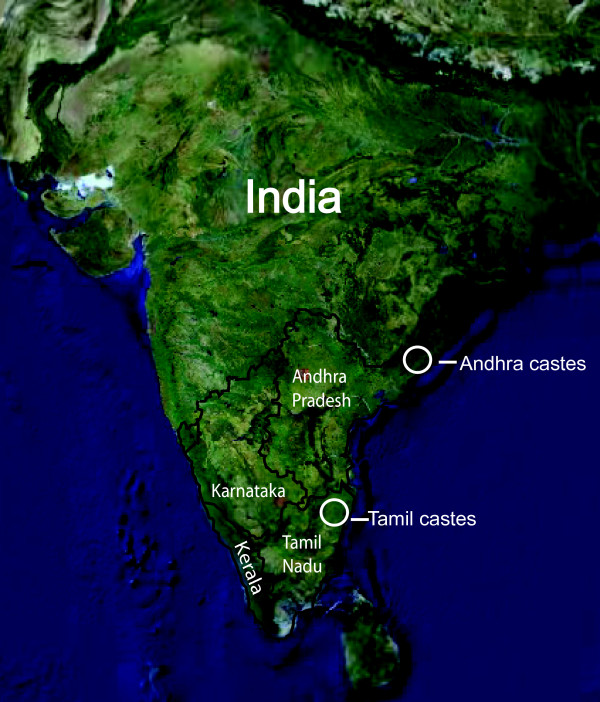
**Map of South India**. A map of the four major states of South India shows the sample locations for the caste populations (figure adapted from Google maps).

We evaluated the genetic relationship between Tamil castes, eastern Asians, and Europeans using 32 lineage-specific Y-chromosome SNPs. The sampling locations for the Tamil castes and a comparative set of castes from the neighboring state of Andhra Pradesh are shown in Figure 1. Y-haplogroups F*, H1, J2/J2a, L1, R1a1, and R2 reach appreciable frequencies (> 5%) in most castes. Common Y-haplogroups are typically shared among castes (Tables 1 and 2). Haplogroup R (predominantly R1a1 (27%) and R2 (11%)) is the most common major lineage in the Tamil castes, followed by H (21%, predominantly H1), L (13%, predominantly L1), J (11%, predominantly J2), and F* (10%).

**Table 1:** Y-chromosome haplogroup frequencies for South Indian castes and major population groups

	Tamil Nadu				Andhra Pradesh			Major Geographic Groups		
Haplogroup	Upper	NTS Upper	Middle	Lower	Upper	Middle	Lower	Europeans	E. Asians	S. Indians
C	0.073	0.027	.	0.029	0.030	0.025	0.056	0.018	0.036	0.034
F*	0.049	0.054	0.140	0.206	0.030	0.050	0.204	.	.	0.103
G	0.024	.	.	.	0.030	.	0.019	.	.	0.009
H	0.098	.	.	.	.	.	0.019	.	.	0.016
H1	0.024	0.189	0.163	0.353	0.152	0.250	0.185	.	.	0.193
I	.	.	.	.	.	.	.	0.316	.	.
J2	0.049	.	0.023	.	0.091	0.100	0.056	0.018	.	0.053
J2a	0.073	0.054	0.140	0.088	.	0.050	.	.	.	0.056
K*	.	0.054	.	.	.	.	.	.	0.071	0.006
L	0.024	.	0.023	0.029	.	0.025	.	.	.	0.016
L1	0.073	0.108	0.209	.	0.061	0.163	0.130	.	.	0.118
O	.	.	.	.	.	.	.	.	0.464	.
O3	.	.	.	.	.	.	0.019	.	0.429	0.003
Q	.	.	.	0.088	.	0.025	0.019	0.018	.	0.019
R1	.	.	.	.	.	.	.	0.035	.	.
R1a1	0.342	0.432	0.186	0.206	0.515	0.188	0.167	0.053	.	0.267
R1b3	.	.	.	.	0.030	.	.	0.544	.	0.003
R2	0.171	0.081	0.116	.	0.061	0.125	0.130	.	.	0.106

**Table 2:** Y-chromosome haplogroup counts for South Indian castes and major population groups

	Tamil Nadu				Andhra Pradesh			Major Geographic Groups		
Haplogroup	Upper	NTS Upper	Middle	Lower	Upper	Middle	Lower	Europeans	E. Asians	S. Indians
C	3	1	0	1	1	2	3	1	1	11
F*	2	2	6	7	1	4	11	0	0	33
G	1	0	0	0	1	0	1	0	0	3
H	4	0	0	0	0	0	1	0	0	5
H1	1	7	7	12	5	20	10	0	0	62
I	0	0	0	0	0	0	0	18	0	0
J2	2	0	1	0	3	8	3	1	0	17
J2a	3	2	6	3	0	4	0	0	0	18
K*	0	2	0	0	0	0	0	0	2	2
L	1	0	1	1	0	2	0	0	0	5
L1	3	4	9	0	2	13	7	0	0	38
O	0	0	0	0	0	0	0	0	13	0
O3	0	0	0	0	0	0	1	0	12	1
Q	0	0	0	3	0	2	1	1	0	6
R1	0	0	0	0	0	0	0	2	0	0
R1a1	14	16	8	7	17	15	9	3	0	86
R1b3	0	0	0	0	1	0	0	31	0	1
R2	7	3	5	0	2	10	7	0	0	34

Total	41	37	43	34	33	80	54	57	28	322

(See Materials and Methods for marker information.)

Some between-caste trends are suggested by the data. The F* lineage is found at higher frequencies in lower castes than in upper or middle castes. The R1a1 lineage occurs at a higher frequency in upper vs. lower castes and differed significantly in frequency in Andhra upper vs. Andhra lower (*p *< 0.05). These trends appear in castes from both Tamil Nadu and Andhra Pradesh. Lineage H also reaches substantial frequency in the Tamil lower caste but is less common in upper and middle castes. Lineage J2, previously shown to be distributed in a northwest to southeast gradient, [3] was present in all castes but not correlated with caste rank.

### mtDNA haplogroups

Tamil castes are characterized by high frequencies of mitochondrial M and N super-family lineages, and all South Indian lineages could be assigned to either M or N clades (Tables 3 and 4). Both major haplogroup super-families are deep-rooting in South Indian populations, with diversity estimates for N (0.01589, n = 63) exceeding that for M (0.01044, n = 92), based on HVS1 data. In contrast to the South Indian mtDNA haplogroup pool, the eastern Asian and European groups have predominantly either M or N lineages, respectively. High diversity and deep-coalescence dates (> 40 K ybp) for both major mtDNA superfamilies are consistent with an ancient and continuous presence of populations in South India that greatly predates the documented history of the caste system.

**Table 3:** mtDNA haplogroup frequencies for South Indian castes and major population groups

	Tamil Nadu				Andhra Pradesh			Major Geographic Groups		
Haplogroup	Upper	NTS Upper	Middle	Lower	Upper	Middle	Lower	Europeans	E. Asians	S. Indians
*M-lineages*										
M*	0.463	0.622	0.442	0.882	0.667	0.600	0.685	.	0.464	0.615
C	0.024	.	.	.	.	.	.	.	0.036	0.003
D	.	.	.	.	.	.	.	.	0.179	.
G	.	.	.	.	.	0.013	.	.	.	0.003
Z	.	.	.	.	.	.	.	.	0.036	.
*M subtotal*	*0.487*	*0.622*	*0.442*	*0.882*	*0.667*	*0.613*	*0.685*	*0*	*0.715*	*0.621*
										
*N-lineages*										
N*	.	.	0.023	.	.	.	0.037	.	0.071	0.009
W	.	.	.	.	.	.	.	0.035	.	.
R	0.024	0.081	0.186	0.029	0.030	0.138	0.074	0.035	0.214	0.090
R5	0.073	.	.	.	0.030	0.013	0.111	.	.	0.034
J	.	.	.	.	.	0.013	.	0.105	.	0.003
T	0.024	.	.	.	.	0.025	0.019	0.088	.	0.012
HV	0.073	0.108	0.047	0.029	0.091	0.063	0.019	0.579	.	0.059
U	0.122	.	.	.	.	.	.	.	.	0.016
U5	.	0.027	.	.	.	.	.	0.018	.	0.003
U5a1	.	0.027	.	.	.	.	.	0.070	.	0.003
U5a/b	.	.	.	.	.	0.013	.	.	.	0.003
U2	0.024	.	.	.	.	.	.	.	.	0.003
U2-K	0.098	0.027	0.209	0.059	0.061	0.100	0.019	.	.	0.084
U4	0.024	.	.	.	.	.	.	.	.	0.003
U7	0.049	0.108	0.093	.	0.091	0.025	0.019	.	.	0.050
U8b	.	.	.	.	.	.	.	0.070	.	.
U9	.	.	.	.	0.030	.	0.019	.	.	0.006
*N subtotal*	*0.511*	*0.378*	*0.558*	*0.117*	*0.333*	*0.390*	*0.317*	*1.000*	*0.285*	*0.378*

(Lineages are grouped by M or N super-family; *could not be further resolved)

**Table 4:** mtDNA haplogroup counts for South Indian castes and major population groups

	Tamil Nadu				Andhra Pradesh			Major Geographic Groups		
Haplogroup	Upper	NTS Upper	Middle	Lower	Upper	Middle	Lower	Europeans	E. Asians	S. Indians
*M-lineages*										
M*	19	23	19	30	22	48	37	0	13	198
C	1	0	0	0	0	0	0	0	1	1
D	0	0	0	0	0	0	0	0	5	0
G	0	0	0	0	0	1	0	0	0	1
Z	0	0	0	0	0	0	0	0	1	0
*N-lineages*										
N*	0	0	1	0	0	0	2	0	2	3
W	0	0	0	0	0	0	0	2	0	0
R	1	3	8	1	1	11	4	2	6	29
R5	3	0	0	0	1	1	6	0	0	11
J	0	0	0	0	0	1	0	6	0	1
T	1	0	0	0	0	2	1	5	0	4
HV	3	4	2	1	3	5	1	33	0	19
U	5	0	0	0	0	0	0	0	0	5
U5	0	1	0	0	0	0	0	1	0	1
U5a1	0	1	0	0	0	0	0	4	0	1
U5a/b	0	0	0	0	0	1	0	0	0	1
U2	1	0	0	0	0	0	0	0	0	1
U2-K	4	1	9	2	2	8	1	0	0	27
U4	1	0	0	0	0	0	0	0	0	1
U7	2	4	4	0	3	2	1	0	0	16
U8b	0	0	0	0	0	0	0	4	0	0
U9	0	0	0	0	1	0	1	0	0	2

Total	41	37	43	34	33	80	54	57	28	322

(Lineages grouped by M or N superfamily; *could not be further resolved; see Materials and Methods for marker information.)

To further examine potential western and central Eurasian contributions to South Indian castes, mitochondrial U lineages, defined by coding variant 12308G, were analyzed in greater detail (Table 5). U haplogroup subtypes were assigned using key HVS1 variants as previously described [4,38]. South Asian lineages U2a and U2c are common in Tamil and Andhra castes. U7 is the most prevalent U lineage in Tamil and Andhra castes. U7 is also common in Iran, Pakistan, and northern India, [39] suggesting an affinity between Dravidian populations from South India and populations to the north and west. A comparison of HVS1 for U7 haplogroups (10) with Indian/Pakistani HVS1 sequences available in the mtDB database (4) revealed similar but non-identical motifs, suggesting ancient rather than very recent gene flow between northwestern and southern India. A notable between-caste difference is observed for the mtDNA haplogroup U data in that the Tamil lower caste sample has a lower frequency of U haplogroups (all subclades) than Tamil upper castes (0.317 vs. 0.059, *p *< 0.05) or middle castes. This trend is also present in the Andhra sample, but it is not significant.

**Table 5:** mtDNA haplogroup U counts for South Indian castes and Europeans

	Tamil Castes				Andhra Castes			Major geographic groups	
	Upper	NTS Upper	Middle	Lower	Upper	Middle	Lower	Europeans	S. Indians
U1a	5	1	1	0	0	0	0	0	7
U2a	1	0	2	0	1	3	0	0	7
U2b	0	0	0	1	0	0	0	0	1
U2c	2	0	4	1	0	4	1	0	12
U2d	0	0	0	0	0	0	0	0	0
U2e	2	0	0	0	0	0	0	0	2
U2i	0	0	2	0	0	0	0	0	2
U4	1	0	0	0	0	0	0	0	1
U5	0	2	0	0	0	1	0	0	3
U5a	0	0	0	0	0	0	0	5	0
U7	2	4	4	0	3	2	1	0	16
K	0	0	0	0	2	1	1	4	4

Total	13	7	13	2	6	11	3	9	55

### Genetic distances

We calculated genetic distances between Tamil castes, Europeans, and East Asians and compared these results to those from upper, middle, and lower caste groups from the neighboring state of Andhra Pradesh. The genetic distance estimates reveal several distinct patterns (Table 6).

**Table 6:** Genetic distance estimates between South Indian castes, Europeans, and eastern Asians

	Y-Chromosome^a^		Autosomal STRs^b^		mtDNA^a^	
	Europeans	E. Asians	Europeans	E. Asians	Europeans	E. Asians
Tamil Upper	0.1730	0.6379	0.0026	0.0067	0.2059	0.0772
NTS Upper	0.1947	0.7094	0.0046	0.0082	0.2640	0.0356
Tamil Middle	0.2737	0.5075	0.0037	0.0077	0.1861	0.0810
Tamil Lower	0.3652	0.7060	0.0052	0.0069	0.5951	0.0383
Andhra Upper	0.1992	0.9352	0.0023	0.0082	0.3124	0.0284
Andhra Middle	0.2527	0.4431	0.0041	0.0064	0.2401	0.0239
Andhra Lower	0.2693	0.4885	0.0053	0.0070	0.3264	0.0154

(^a^Slatkin's linearized F_ST _distance; ^b^D_SW _distance)

For Y-chromosome polymorphisms, all castes have smaller distances to Europeans than to eastern Asians. For mtDNA polymorphisms, all castes have smaller distance estimates to eastern Asians than to Europeans. For Y-chromosome data, the genetic distance estimates to the Europeans is ordered by caste rank. These trends appear in castes from both geographic regions.

**Figure 2:**
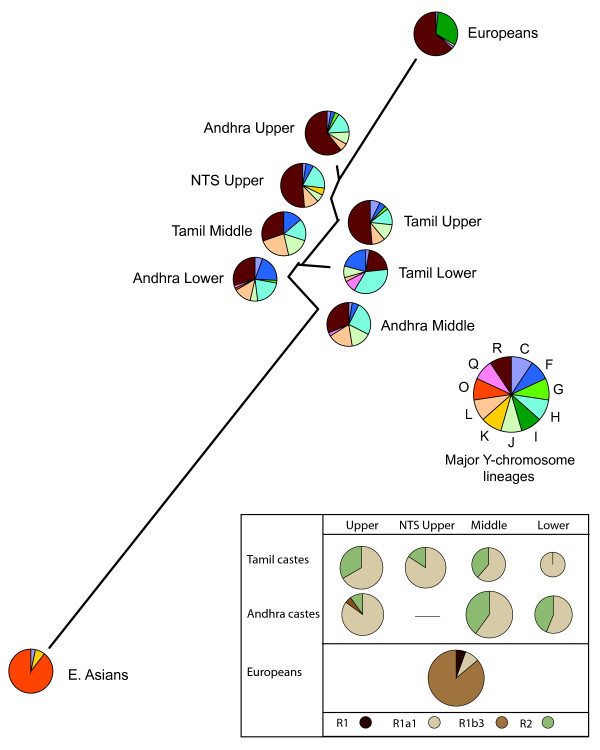
**Genetic distances for Y-chromosome data**. A neighbor-joining network depicts the genetic distance estimates between South Indian castes, Europeans, and East Asians for 32 Y-chromosome SNPs. The pie diagrams indicate the proportion of each major Y-chromosome lineage found in each population. The inset shows the proportions of Y-chromosome R sub-lineages. Inset circle size is proportional to the total number of R lineages.

A neighbor-joining network depicts the between-population relationships based on Y-chromosome data (Figure 2). The NTS Upper caste is more closely related to the Andhra Upper caste than to the other Tamil castes, a finding consistent with a common language (Telugu) shared by the NTS Upper and Andhra upper castes. All castes are closer to Europeans than to eastern Asians, and basal haplogroup R is common, especially in the upper castes and Europeans. The inset, however, shows that haplogroups derived from R are not commonly shared between this sample of Europeans and southern Indians. Affinity between the groups is driven largely by basal characters (R, F* and H) that have contrasting frequency patterns.

**Figure 3:**
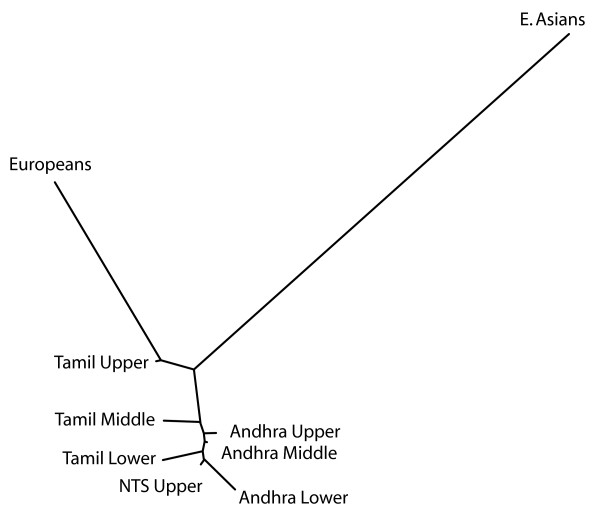
**Genetic distances for autosomal data**. A neighbor-joining network depicts the genetic distance estimates (D_SW_) between South Indian castes, Europeans, and East Asians for 45 autosomal STRs.

A neighbor-joining network based on distance estimates from 45 STRs shows a greater affinity of all castes to Europeans than to eastern Asians (Figure 3). With the exception of the NTS Upper (Telugu and Kannada speaking) Brahmins, castes of similar rank from different geographic locations tend to branch at similar locations within the network. Within each geographic region, the distances to other Eurasians (both Europeans and East Asians) increases with decreasing caste rank.

**Figure 4:**
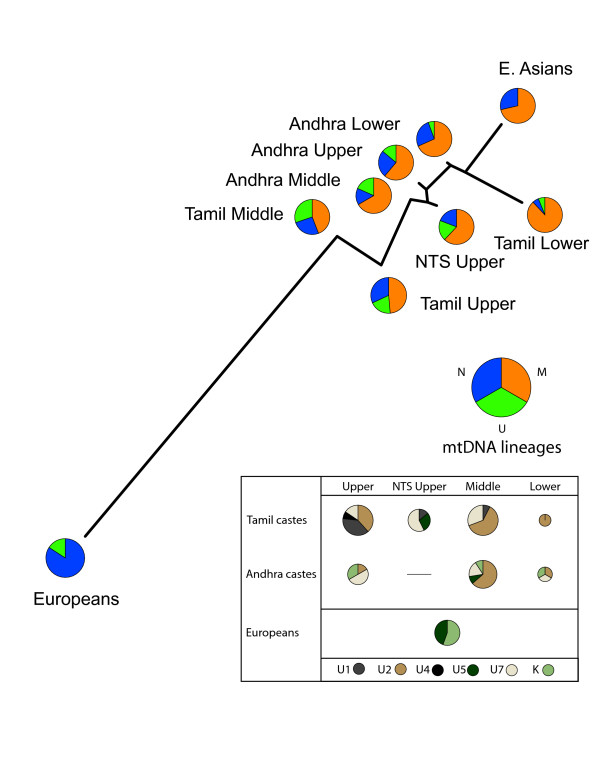
**Genetic distances for mitochrondrial data**. A neighbor-joining network depicts the genetic distance estimates between South Indian castes, Europeans, and East Asians for 32 mtDNA SNPs and 411 bp of HVS1 sequence. Pie diagrams indicate the proportion of major mtDNA lineages found in each population. The inset shows the proportion of mtDNA U sub-lineages. Inset circle size is proportional to the total number of U lineages.

The network based on mitochondrial distance estimates shows little between-caste rank organization, yet reveals the greater affinity of all castes to eastern Asians for maternal lineages (Figure 4). Basal U haplogroups are less frequent in lower rank castes from both southern India locations. The inset shows that only a few high-resolution U haplogroups (U5, K) are shared between Europeans and South Indians.

### Genetic structure

The proportion of genetic variation distributed within and between South Indian castes was assessed by an analysis of molecular variance (AMOVA) (Table 7). The Tamil South Indian castes are only modestly differentiated from one another: 0.96% of STR variance occurs between Tamil castes. A similar value of 0.77% for between-population (caste) difference is observed in the Andhra castes. A smaller fraction, 0.12%, is attributable to geographic differences between Tamil and Andhra locations and was not significantly different from zero. Removal of the NTS Upper caste from the comparison yielded a non-significant but higher value of 0.28%. These findings, based on multiple unlinked loci, suggest that social structure has had a larger impact on caste population structure in these South Indian samples than geographic separation.

**Table 7:** AMOVA for Y-chromosome, 45 autosomal STRs, and mtDNA

	Tamil Castes	Andhra Castes	South Indians
	*(4 castes)*	*(3 castes)*	*(2 states, 7 castes)*
Y-chromosome			
*Among states*	-	-	-1.88^a^
*Among castes*	4.45	5.52	5.06
*Within castes*	95.55	94.48	96.82
			
Autosomal STRs			
*Among states*	-	-	0.12^a, b^
*Among castes*	0.96	0.77	0.85
*Within castes*	99.04	99.23	99.02
			
mtDNA			
*Among states*	-	-	-0.47^a^
*Among castes*	4.50	0.17^a^	2.41
*Within castes*	95.50	99.83	98.06

(Values are shown as percentages. All values differ significantly from a null distribution (*p *< 0.05) unless indicated by [^a^]. ^b^This value increases to 0.28 without the NTS Upper caste but remains non-significant. Tamil castes: Upper, NTS Upper, Middle, Lower; Andhra castes: Upper, Middle, Lower)

Y-chromosome and mtDNA estimates of molecular variance between castes samples from either Tamil Nadu or Andhra Pradesh also exceed the estimate for between-group variation for the two geographic regions. Between-caste variation for mtDNA in Tamil populations is greater than that for Andhra populations. This may be partly due to regionally high female mobility in Andhra castes as previously reported [20,30]. As expected, for all genetic systems, the vast majority of all variation occurs *within *populations.

The degree of population subdivision among Indian castes was estimated using a model-based clustering method implemented in STRUCTURE (ver. 2.1). The best estimate of the number of clusters (K) was consistently one for the Tamil Indians. The best estimate of the K clusters was also one for Tamil and Andhra castes together. This result indicates that individuals from castes spanning the Indian social hierarchy from two independent geographic regions are not sufficiently differentiated to allow clustering into groups based on genetic data from 45 STR polymorphisms alone. This finding is consistent with the low R_ST _values for these populations but may also reflect the limited power of 45 STRs to distinguish such closely related populations. Estimates for heterozygosity and repeat variance in these populations also indicate no substantial between-caste differences or excess homozygosity in these caste groups (Table 8).

**Table 8:** STR heterozygosity and variance for South Indian caste populations

	H_exp_	H_obs_	Alleles per locus^a^	Variance^a^
Tamil Upper	0.728 (+/- 0.020)	0.700	7.1	3.4
NTS Upper	0.716 (+/- 0.019)	0.683	6.6	3.2
Tamil Middle	0.718 (+/- 0.020)	0.681	6.8	3.3
Tamil Lower	0.716 (+/- 0.021)	0.699	6.4	3.3
Andhra Upper	0.732 (+/- 0.018)	0.708	6.7	3.6
Andhra Middle	0.721 (+/- 0.020)	0.693	7.6	3.4
Andhra Lower	0.719 (+/- 0.020)	0.704	7.5	3.4
E. Asians	0.692 (+/- 0.025)	0.697	6.5	3.4
Europeans	0.716 (+/- 0.017)	0.695	7.3	3.2

(^a^Average variance in repeat number over 45 STR loci)

We evaluated the correlation between caste rank and genetic distance using a Mantel test (Table 9). For each test, a correlation between pairwise genetic and pairwise caste rank distances matrices using the Tamil caste individuals was calculated. For Tamil-speaking populations, all genetic systems produced low, significant positive correlations. Y-chromosome haplogroup data yielded the highest positive correlation with caste rank (*ρ *= 0.26, *p *< 0.01). Inclusion of the non-Tamil speaking Brahmins decreases the correlation for all systems.

**Table 9:** Spearman's correlation between genetic distance and caste rank.

	Tamil – speaking castes	All Tamil castes
Y-chromosome	0.26*	0.15*
45 STRs	0.06*	0.03
mtDNA	0.10*	0.06*

(* *p *< 0.01)

## Discussion

Using a geographically well-defined sample of caste populations from Tamil Nadu, India, this study arrives at many conclusions similar to those from our previous studies of caste populations from Andhra Pradesh, India [13,20,30]. In both cases, there is extensive sharing of Y and mtDNA haplogroups among castes, and the overall level of inter-caste differentiation is low. This finding is consistent with many other studies of genetic structure and gene flow patterns among caste populations [6,32,33,40].

Paternally-inherited Y-chromosome SNPs show that caste populations have greater affinity to a sample of Europeans than to a sample of eastern Asians. Unlike the Y-chromosome data, maternally-inherited mtDNA polymorphisms demonstrate a contrasting pattern – castes, regardless of rank, have higher affinity to eastern Asians than to Europeans. These patterns were present in samples from both geographical locations suggesting that South Indian paternal lineages have been more substantially influenced by western or central Eurasians compared to South Indian maternal lineages. Unlike our previous study of Andhra castes, [13] direct haplogroup sharing between Tamil castes and our sample of Europeans is more limited, suggesting a potentially greater time depth for the development of these patterns. More extensive sampling will be required to resolve this difference.

Using Y-chromosome data, Tamil castes of different rank have differential affinities to our sample of Europeans, with upper castes demonstrating greater affinity than lower castes. Genetic distances are weakly correlated with caste rank distances and correlations from Y-chromosome data are stronger than correlations based on mtDNA or autosomal data. This pattern argues for a differential contribution of male lineages to castes of different rank and limited male mobility between castes in South India.

An interesting difference between the data sets from Andhra Pradesh and Tamil Nadu is also observed. For the former sample, inter-caste distance based on mtDNA polymorphisms (HVS1 sequence) demonstrated a strong relationship to caste rank, while distances based on Y-chromosome data did not. This was interpreted as evidence of historical upward female mobility in the caste system [30]. (We note, however, that the primary reason for a lack of correlation between Y-chromosome distances and caste rank was close affinity between the upper-caste Brahmin and lower-caste Relli samples [20].) In contrast, the Tamil Nadu samples show a higher correlation between Y-chromosome distances and caste rank than between mtDNA distances and caste rank. This difference likely reflects differential apportioning of individuals as the caste system originated or subsequent differences in male-female mobility patterns.

Recently, several studies have underscored the complexity of Y-chromosome variation in Indian populations. Sahoo et al. (2006) presented evidence that the R1a haplogroup has attained high frequencies and high diversity in northern India, central Asia, and eastern Europe. They also reported high frequencies of Y-chromosome haplogroup H in caste and tribal populations and provided compelling evidence for an origin of haplogroup H in South India. Upon further analysis, their data show that, as in our study, the frequency of haplogroup R lineages is higher in Brahmins (upper rank) than in lower-rank castes (0.53 vs. 0.41), while the frequency of H lineages is lower in Brahmins than in lower castes (0.15 vs. 0.34).

In a study of broadly distributed Indo-European and Dravidian castes, Sengupta et al. (2006) suggested that the majority of Indian Y-chromosome haplogroups are at least 10,000 to 15,000 years old as gauged by Y-chromosome microsatellite diversity, thus predating the origin of the caste system. The antiquity and complex geographic distribution of the R1a1 and R2 haplogroups led these authors to conclude that the majority of the subcontinent Y-chromosomes arrived in or before the early Holocene (10,000 years ago) rather than in a later Indo-European expansion. Likewise, and concordant with other studies of tribal Indian populations, [5] we observe Y-chromosome R1a1 lineages in South Indian tribal Irula (unpublished data), a population substantially differentiated from South Indian castes [18].

An examination of the R and H haplogroup frequencies of Indo-European-speaking castes reported by Sengupta et al. (2006) shows that, as in our study, R haplogroup frequencies in upper castes exceeded those of middle and lower castes (0.62, 0.35, and 0.38, respectively), while H haplogroup frequencies were lowest in upper castes (0.14), intermediate in middle castes (0.38), and most frequent in lower castes (0.44). For Dravidian castes, R (0.62) was more frequent than H (0.14) in upper castes while R and H had similar (within 6%) frequencies in middle and lower castes.

A recent analysis of caste and tribal populations from eastern India (Orissa) demonstrated Indo-European influences on paternal caste lineages [41]. Brahmins showed high Y-chromosome affinity to eastern Europeans (M17, haplogroup R1a1). In contrast, maternal mtDNA polymorphisms revealed primarily Indian-specific lineages. Taken together, our studies and at least three other studies of Y-chromosome lineages in Indian castes demonstrate that upper castes show genetic affinity to populations residing north and northwest of the Indian subcontinent. This affinity appears, in part, to result from varying frequencies of Y-chromosome R lineages and older South Asian lineages such as F* and H.

Indian mtDNA lineages demonstrate high diversity, suggesting that a majority of Indian maternal lineages are also relatively old and likely predate historically documented expansion events [38,42]. Older, deep-rooting mitochondrial lineages belonging to the N macrolineage are prevalent in western Eurasia and are distributed in a West – East cline, with high frequencies in Anatolia and Iran and moderate frequencies in Pakistan and northwestern India [43]. In this study we observe higher frequencies of basal U lineages in upper castes than in lower castes. Higher resolution haplogroup results, however, show little evidence of between caste differences. This may indicate differences in founding populations. More likely, though, it may suggest ancient migration and integration of various U haplogroups into different pre-caste populations with subsequent, non-uniform lineage sorting and differentiation over time. In contrast, and consistent with early human expansion across South Asia, the predominantly Asian M clade mitochondrial haplogroups account for more than half of all Indian mitochondrial lineages and reach their highest frequencies in lower caste and tribal groups [6,13].

While Y-chromosome and mtDNA polymorphisms yield valuable information, it must be borne in mind that they each represent a single linkage group. Estimates based on these systems are thus subject to a high level of stochastic variability [44,45]. In addition, the Y-chromosome and mtDNA may both have been affected by natural selection, [46,47] which can further complicate the interpretation of population history. Coalescence dates based on these systems must also be viewed with appropriate caution, in part because of their large confidence intervals. More importantly, a coalescence date is not necessarily a reliable indicator of the founding date of a population [45] because these dates are affected by the size of the founder population and by subsequent gene flow patterns. To gain a more complete and reliable portrait of population history, multiple, independent autosomal polymorphisms should also be examined.

Our analysis of 45 unlinked autosomal STRs reveals that in Tamil Nadu, genetic distances between castes are positively correlated with caste rank. A similar pattern was detected in upper, middle, and lower rank castes of Andhra Pradesh using these STRs [20] and *Alu *and L1 insertion polymorphisms [13]. An analysis of the Kallar, Vanniyar, and Pallar castes, which also reside in Tamil Nadu, showed that upper – lower caste distance estimates (0.0553) exceeded those for upper – middle castes (0.0329) and middle – lower castes (0.0515) [40]. Majumder et al. [37,48] presented Y-chromosome, mtDNA, and autosomal data from several caste populations in Uttar Pradesh. Subsequent analysis indicated that caste rank was correlated with genetic distance for all three types of systems [20]. Similar correlations have been observed in a number of other studies of Indian populations [31,33,49]. A relatively greater affinity between upper-caste populations and Europeans has been observed for autosomal polymorphisms in our Andhra Pradesh and Tamil Nadu samples and in a number of other analyses of autosomal data [6,50,51].

Although significant correlations between caste-rank and genetic distances are apparent, model-based clustering algorithms did not detect structure within the Tamil or Andhra populations. We suggest that this finding results from the low amount of differentiation between all caste groups but also from a lack of sufficient power in 45 unlinked STRs to detect high-resolution population structure. With ~250 K SNPs typed in a subset of the Andhra upper and Andhra lower castes, individuals can be clustered into these population groups using genotype information alone [52]. Likewise, using > 950 K SNPs, the Tamil upper and Tamil lower castes demonstrate group-specific clustering by principal component analysis (unpublished data).

Considering the complex history of Indian populations, it is not surprising that some studies demonstrate an association between caste rank and genetic distance, whereas others do not. A recent study of 15 geographically dispersed Indian populations residing in the United States using 1200 markers found little evidence for caste or geographic structure [53]. However, sampling strategy (relocated vs. *in situ*) or other factors, such as a very wide geographic dispersion of the study populations, may confound correlations if they exist. Admixture and gene flow can also vary substantially between caste populations in the various regions of India. Linguistic differences may influence the genetic structure of local caste populations [34]. The linguistically different NTS Upper caste Brahmins showed several differences in comparison to the other Tamil castes in this analysis. Yet, because Indian populations show only a small amount of genetic differentiation, [17,53] a large number of autosomal loci will be necessary for adequate power to detect consistent patterns of variation if they are present [54,55]. Ancestry-informative autosomal polymorphisms, high-density genotyping, and extensive population sampling will provide better resolution of the relationships between Indian and other Eurasian populations.

The results presented here underscore the complexity of the Indian caste system. Although other interpretations may be possible, our data are consistent with a model in which nomadic populations from northwest and central Eurasia intercalated over millennia into an already complex, genetically diverse set of subcontinental populations. As these populations grew, mixed, and expanded, a system of social stratification likely developed *in situ*, spreading to the Indo-Gangetic plain, and then southward over the Deccan plateau. A strong patrilineal social structure, accompanied by a developing practice of caste endogamy, may have contributed to an asymmetric apportioning of Y-chromosome, autosomal, and to a lesser extent, mtDNA lineages. Remnants of these patterns can still be detected in some of the inhabitants of peninsular South India.

## Conclusion

Genetic variation between South Indian castes from Tamil Nadu is low (R_ST _= 0.0096). Tamil caste Y-chromosomes and STR alleles are more similar to Europeans than to eastern Asians, and genetic distance estimates to Europeans are ordered by caste rank. In contrast, Tamil caste mtDNA shows greater similarity to eastern Asians than to Europeans. Low, but statistically significant, correlations between genetic distance and caste rank can be demonstrated for the Tamil-speaking populations. These patterns likely reflect asymmetric influences of ancient and historical processes on the caste system as it developed. These findings provide a general replication of our analysis of ranked castes from the neighboring state of Andhra Pradesh, India [13]. For the caste populations analyzed here, between-caste genetic differentiation exceeds that due to geographic (between-state) differentiation, a finding that may be of considerable interest when initiating linkage mapping [56] and case-control association studies in South Indian populations.

## Methods

### Study Subjects

Study subjects were recruited from four caste groups in Tamil Nadu, India. Tamil-speaking Brahmins (41), Mudaliars (43), and Dalits (Harijans) (34) were sampled in Chennai or from rural locations near Chennai. Caste rank was assigned using the traditional varna of Brahmin (Brahmin, upper ranking), Mudaliar (Sudra, middle ranking), and Dalit (scheduled caste – outside the traditional caste system, lower ranking). A second sampling of Brahmins (37) was obtained in Kanchipuram, located ~70 km southwest of Chennai. The Kanchipuram Brahmin group is linguistically diverse, containing Kannada- and Telugu-speaking Brahmins that relocated from the neighboring states of Andhra Pradesh and Karnataka. This group of upper castes individuals is referred to subsequently as the non-Tamil speaking (NTS) Upper caste. This study was approved by the Schizophrenia Research Foundation, Chennai, India and by the Wolston Park Hospital Ethics Committee, Brisbane. Approvals were also obtained from the Indian Council of Medical Research and the Indian Ministry of Commerce. Written, informed consent was obtained from all participants.

A comparative European sample of northern European and French ancestry (57), and eastern Asians of Chinese, Japanese, and S.E. Asian ancestry (28) have been previously described [28,57,58]. Because all samples were required to have data for all genetic systems thus excluding females, sample sizes are smaller than previously reported. The comparative sample of populations from Andhra Pradesh, India includes upper-caste Brahmins (33), middle-caste Kapus and Yadavas (80), and lower-caste Malas, Madigas, and Rellis (54) [13].

### Data collection

DNA was extracted from venous blood using standard procedures. Hypervariable sequence 1 (HVS1), corresponding to base pairs 16000 – 16410, was amplified by PCR and sequenced using BigDye 3.1 dye-terminator fluorescent sequencing chemistry and an Applied Biosystems (ABI) 3100 automated sequencer.

Lineage and sub-lineage identifying single nucleotide polymorphisms (SNPs) for the mitochondria (32 markers) and Y-chromosome (32 markers) were selected from the literature [47,59-64]. Lineage-defining mitochondrial coding region markers used in the study are L2-C10810T, M-C10400T, C-A13263G, D-C4883T, preE-G4491A, E-G7598A, G-A4833G, Z-T9090C, N-C10873T, N1d-C6713T, Y-A7933G, W-G8994A, R-T12705C, R5-T8594C, J-A12612G, T-T10463C, H/V-T14766C, U-A12308G, U6-A3348G, U6a-G7805A, U5-T3197C, U5a1-A14793G, U5a/b-A7768G, U2-K-A1811G, U2-A3720G, U2-A9545G, U3-G9266A, U4-T4646C, U7-C5360T, U7-C8137T, K/U8-G9055A, and U9-G3531A. Y-chromosome lineages and markers used are C-M216, F*-M89, G-M201, H-M52, H1-M82, H1a-M36, H1b-M97, H1c-M138, I-M170, J2-M172, J2a-M410, K*-M9, K1-SRY9138, K2-M70, L-M20, L1-M76, M-M5, N-LLY22g, O-M175, O3-M122, P*-M74, Q-P36, Q3-M3, R*-M207, R1-M173, R1a-SRY10831.2, R1a1-M17, R1a1a-M56, R1a1b-M157, R1a1c-M87, R1b3-M269, and R2-M124.

Mitochondrial and Y-chromosome SNPs were genotyped by fluorescent primer extension using SNaPshot chemistry (ABI). Primers were annealed to amplification products adjacent to the polymorphic site and extended by one nucleotide using the manufacturer's recommendations. Extension products were pooled and resolved on a 36-cm capillary array. Four to eight SNPs were assayed per multiplex. Forty-five STRs, predominantly tetranucleotide repeats, were amplified using 5'-NED, -PET, -VIC, or -6-FAM labeled primers using standard PCR conditions and resolved in 5 fluorescent multiplex runs on an ABI 3100. STR loci are UT1091, UT1201, UT1205, UT1220, UT1227, UT1228, UT1232, UT1239, UT1243, UT1257, UT1313, UT1352, UT1357, UT1376, UT1674, UT1708, UT1740, UT1747, UT1880, UT1885, UT1917, UT1950, UT1985, UT2021, UT2081, UT2092, UT2127, UT2203, UT5022, UT5027, UT5029, UT5030, UT5033, UT5048, UT5492, UT6507, UT6516, UT6540, UT7131, UT8067, UT868, UT871, UT901, UT919, and vWFII. These STRs and mtDNA polymorphisms, were typed in comparative populations as described previously [13,18,20,57,58]. Y-chromosome, STR, and mtDNA genotype data is provided in the Additional_file 1.

To allow a direct comparison of Y-chromosome haplogroups from Tamil Nadu castes to those from Andhra Pradesh castes, we typed individuals from Andhra Pradesh for 26 of the 32 lineage-defining SNPs. A Y-haplogroup was assigned to each sample by the presence of one or more derived-state alleles, and the remaining alleles were inferred. This SNP panel allowed further refinement of the haplogroups previously reported for the Andhra Pradesh samples [13,30].

### Data analysis

Haplogroups for the Y-chromosome (32 SNPs) and mtDNA (32 SNPs and 411bp HVS1 sequence) were assigned using SNP data. Mitochondrial haplogroups were assigned to a haplogroup based on the most probable consensus of polymorphic changes or resolved using previously published mtDNA HVS1 motifs as a guide [62]. Thirty-one exceptions to the canonical mtDNA phylogeny occurred on 27 mtDNA haplogroups, and these haplogroups with recurrent mutations were assigned to the most likely haplogroup based on HVS1 sequence data [4,6]. The variant 7598A, defining mtDNA lineage M-E, was found in 2 Tamil and 1 Andhra individuals who share identical HVS1 motifs but lack the preE 4491A variant. Between-caste haplogroup differences were evaluated for significance using Fisher's exact test.

Diversity estimates (F_ST_, R_ST_, and AMOVA) for Y-chromosome, mtDNA, and autosomal STRs were calculated using the ARLEQUIN 3.0 software package [65]. AMOVA statistics were evaluated for significance by comparison to an empirical distribution generated by random permutation of genotypes or haplogroups. A general age estimate for mtDNA coalescent dates was calculated by the method of Nei [66] using a substitution rate of 2 × 10^-7 ^substitutions/site/year [67].

Model-based analyses of population structure were performed using the STRUCTURE program [68]. An estimate of the optimal number of clusters (K) for the four Tamil castes was obtained from the posterior probabilities of K, P(X|K), averaged over 10 runs for each value of K. A uniform prior probability distribution was assumed on K = {1...*n*}, and burn-in and iterations were set to 10,000 each for estimating the best K. Estimates of proportionate membership to three clusters were averaged values from 10 independent STRUCTURE runs. Population admixture and correlated allele frequencies were used in all analyses.

The correlation between genetic distance and caste rank was assessed by Mantel matrix tests using Spearman's rank correlation. For all possible pairs of caste individuals, inter-individual genetic distance estimates were calculated using DNADIST (Y and mtDNA) [69] or the D_sw _program (STRs) [70]. Next, each individual was assigned a ranking (1, 2, or 3) for upper, middle, and lower caste status. The difference in caste rank was calculated for all possible pairs of caste individuals, yielding a full pair-wise matrix (155 × 155, or 118 × 118 for Tamil-speakers only) of ordinal values (0, 1, 2). Spearman's rank correlation between the genetic distance (Y-chromosome, mtDNA, or autosomal STRs) matrix and the caste rank difference matrix was calculated. A significance level for the correlation was determined by comparing the actual correlation to a distribution of correlations generated by 10,000 random columnar permutations.

## Authors' contributions

WSW carried out the molecular studies, performed data analysis, and drafted the manuscript. RT performed analysis and sample collection in India. BJM and DN designed and partially funded the study. YZ performed genotyping and laboratory experiments. DJW provided statistical consultation. WT performed genotyping and other laboratory experiments. MJB helped acquire and analyze samples from Andhra Pradesh. ST and RP performed sample collection in Tamil Nadu. HS and CF performed sample extraction and laboratory analysis of samples from Tamil Nadu. LBJ designed, coordinated, and funded the study. All authors read and approved the final manuscript.

## Supplementary Material

Additional file 1 **Y-chromosome, STR, and mtDNA genotype data.** Genotype data for South Indians, Europeans, and eastern Asians. Click here for file
